# Editorial for the Special Issue on Polymer Based MEMS and Microfabrication

**DOI:** 10.3390/mi10010049

**Published:** 2019-01-11

**Authors:** Dan Sameoto

**Affiliations:** Department of Mechanical Engineering, University of Alberta, Edmonton, AB T6G 2G8, Canada

Polymers are becoming increasingly important in MEMS and microfabricated products. Their exceptionally wide range of mechanical, optical, electrical and chemical properties makes their inclusion in applications ranging from sensors, microfluidics and molded bioinspired surfaces a worthwhile endeavor. While polymers will never replace more traditional materials like silicon and metals in large scale MEMS production, their strengths lie in their potential applications in biological systems, rapid prototyping, robust operations, or manufacturing possibilities outside a cleanroom environment. Polymer based microfabrication can enable tolerance to environments that MEMS and microelectronics have frequently avoided. This collection of papers, focusing on Polymer Based MEMS and Microfabrication, includes two review articles and nine original research articles. In the reviews of specific sub-disciplines, Ortigoza-Diaz et al. cover the state of the art use of Parylene C within MEMS systems [1]. The specific technical challenges in microfabrication, including deposition, patterning and bonding with this unique polymer are discussed, along with exemplary demonstrations of its application in biological applications with patterned metal layers, including neural probes, pressure sensors, antennas and others. Several key challenges left to be addressed for improved reliability are identified by the authors as guides to future work by groups working with the material and its interface to biological systems. The second review article by Thuau et al. covers a range of electromechanical transduction schemes for use in polymer based MEMS sensors developed by their group and others [2]. Highlighted devices and materials include piezoresistive composites based on carbon nanotubes, piezoelectric polymer sensors made of PVDF-TrFE, and organic field effect transistors (OFETs). These polymer-based transducers are primarily designed for sensing applications and demonstrate that composite and polymer MEMS can be manufactured and integrated for complex functions.

For the original research articles published in this special issue, polymers are shown to be functional in the most demanding of applications and also exceptionally useful for frugal engineering or rapid prototyping. We have three articles that focus on specific manufacturing improvements that aid in the fabrication of low-cost polymer microfluidics [3,4,5]. Song et al. [3] demonstrate a new technique to bond two dissimilar thermoplastics, polycarbonate (PC) and polymethylmethacrylate (PMMA) via a PMMA solution dissolved in propylene glycol monomethyl ether acetate (PGMEA). In this technique, the attractive machining properties of PMMA for microchannels are integrated with PC which can have higher toughness and superior mechanical and thermal properties and bond strength has been demonstrated up to approximately 700 kPa. Strike et al. [4] also work with PMMA for microfluidics, and in their work, they introduce a new fabrication process with CO_2_ laser micromachining that can both produce channels, as well as weld thermoplastic polyurethane membranes onto PMMA with high density polyethylene release layers for valving and pumping purposes. The integration technique is relatively simple, has very low cost materials like commercial acrylic, tape and food wrap, and was demonstrated to be functional for a simple application of two-phase water in oil droplet generation, with a fabrication time of a few hours. Zhou et al. [5] provides an alternative manufacturing technique for PMMA in microfluidics as high precision milling of a PMMA master mold is used, which can then be subsequently replicated to make polydimethylsiloxane microfluidic systems. Much smaller channels can be produced using a large milling tool when making a positive relief than if it was to mill out channels directly, and the roughness and minimum features were tested before and after PDMS replication to determine tolerances and fidelity. Just as in Strike, an oil-in-water droplet generation system was used to demonstrate functionality of the proposed process. 

Two flexible arrays developed for measuring pressure [6] or providing electrical signals to cochlear implants [7] are reported. In the case of cochlear implants, Parylene-C again is the polymer of choice, but in this work it is reinforced with Kapton to aid in insertion of sensors into tissues. For Liu’s reported sensor [6], aligned multi-walled carbon nanotubes in a polyurethane matrix are electrospun to produce an artificial skin. Unidirectional piezoresistivity can be measured when the sub-micron composite fibers are pressed between two electrodes. The resulting sensor can be easily bent due to the small thicknesses of all layers involved. While resistivity is still very high, future developments with more conductive fibers could lead to cost effective pressure sensitive skins for robots or prosthetics. Zhang et al. [8] present a new etching recipe for Parylene-C based on the addition of a small amount of SF_6_ to an O_2_ plasma that results in a cleaner etching process with less residuals from micromasking. The procedure offers new design parameters for developing biocompatible probes, microfluidics, and more from Parylene, and adds to the material covered in the review by Ortigoza-Diaz [1]. 

Two extremes of polymer microfabrication with 3D printing technologies are demonstrated in separate works by Lamperska et al. [9] and Kundu et al. [10]. Lamperska has used two-photon lithography—the current state of the art 3D polymer nanofabrication technique–to create micro-dumbbell structures that may be manipulated by optical tweezers. The approach allows indirect handing of sensitive structures, or direct application of physical probing of objects in liquids without the influence of optical tweezer light on the object of interest. Kundu has applied more macroscale methods for 3D printing and combined them with ink casting and lamination to provide a rapid prototyping process for applications ranging from microfluidics, micro-electrode arrays, and microneedles. Kundu’s work highlights how the capabilities of “makerspace” style manufacturing methods are beginning to converge, or replace in some instances, the high-cost, high-precision realm of cleanroom-based microfabrication for a variety of uses. 

Finally, my own group reports the use of Fluorosilicone rubber as mold material that can be used in a variety of soft-lithography applications [11]. Fluorosilicone is rarely used in academic literature, but combines high temperature tolerances, good strength and elasticity, and low surface energy to produce a flexible mold that is useful for gecko-inspired adhesives, among other applications. Silicone rubbers can be cast, thermally cured and demolded from these Fluorosilicones in as little as a minute, although exact performance is material specific. Fluorosilicone helps address a major challenge with soft lithography, which is the ability to upscale manufacturing processes when curing materials are used. By curing at very elevated temperatures, and being quite robust compared with rigid microstructured molds, more throughput may be possible in future soft lithography applications.

I would like to thank all the authors of submitted papers to this special issue for making it a success, and the dedication of the many reviewers that put in their time and effort in ensuring the quality and high standards of the papers we have had published. It has been a pleasure working with the staff of Micromachines to make this special issue happen and I appreciate the opportunity to work with you all.
